# Immune‐related adverse events of antibody‐based biological medicines in cancer therapy

**DOI:** 10.1111/jcmm.18470

**Published:** 2024-07-04

**Authors:** Deepa Rajagopal, Elliot MacLeod, Diana Corogeanu, Sandrine Vessillier

**Affiliations:** ^1^ Immunotherapy, Biotherapeutics and Advanced Therapies Division, Science, Research, and Innovation Group, Medicines and Healthcare products Regulatory Agency (MHRA) Hertfordshire UK; ^2^ Present address: Gilead Sciences, Winchester House Oxford UK; ^3^ Present address: East Sussex Healthcare NHS Trust, Conquest Hospital East Sussex UK

**Keywords:** cancer, cytokine release syndrome, harmonisation, immune‐related adverse events, immunotherapies

## Abstract

Recombinant antibodies (Abs) are an integral modality for the treatment of multiple tumour malignancies. Since the Food and Drug Administration (FDA) approval of rituximab as the first monoclonal antibody (mAb) for cancer treatment, several mAbs and antibody (Ab)‐based therapies have been approved for the treatment of solid tumour malignancies and other cancers. These Abs function by either blocking oncogenic pathways or angiogenesis, modulating immune response, or by delivering a conjugated drug. The use of Ab‐based therapy in cancer patients who could benefit from the treatment, however, is still limited by associated toxicity profiles which may stem from biological features and processes related to target binding, alongside biochemical and/or biophysical characteristics of the therapeutic Ab. A significant immune‐related adverse event (irAE) associated with Ab‐based therapies is cytokine release syndrome (CRS), characterized by the development of fever, rash and even marked, life‐threatening hypotension, and acute inflammation with secondary to systemic uncontrolled increase in a range of pro‐inflammatory cytokines. Here, we review irAEs associated with specific classes of approved, Ab‐based novel cancer immunotherapeutics, namely immune checkpoint (IC)‐targeting Abs, bispecific Abs (BsAbs) and Ab‐drug‐conjugates (ADCs), highlighting the significance of harmonization in preclinical assay development for safety assessment of Ab‐based biotherapeutics as an approach to support and refine clinical translation.

## 1 INTRODUCTION

Immunotherapy has revolutionized clinical recovery from several cancer types, providing durable responses in subsets of previously untreatable advanced cancer patients and is developing as a new pillar of cancer therapy. Many approved and emerging antibody (Ab)‐based oncological therapies target immune cell surface markers that function either as receptors, modulators or soluble mediators.

Immune checkpoint (IC)‐inhibiting antibodies (Abs) are the most widely used class of immunostimulatory Abs, which release ‘protein brakes’ on the immune system and reinvigorate a T‐cell‐mediated anti‐tumour response. Additional classes of Ab‐based biotherapeutics emerging as promising oncological treatments include bispecific Abs (BsAbs) and Ab‐drug‐conjugates (ADCs). Clinical success is however limited due to the development of severe immune‐related adverse events (irAEs) reported from clinical trials, highlighting the crucial need to address safety aspects to minimize the risk of serious and potentially life‐threatening complications. While our understanding of underlying mechanisms leading to adverse events (AEs) in patient subsets following treatment is still evolving, further focus on the identification of predictive markers associated with the severity of adverse responses in patients could facilitate safer delivery of treatment.

Here, we review currently approved Ab‐based cancer immunotherapeutics, focussing on IC‐inhibiting Abs, BsAbs and ADCs, in the context of associated irAE.

## 2 IC INHIBITION (ICI) THERAPY

ICI aims to generate anti‐tumour responses by targeting IC proteins, and augmenting tumour‐directed T‐cell responses. IC proteins tightly control immune system dynamics, modulate T‐cell activity and prevent self‐reactive T‐cell‐mediated autoimmunity. Widely studied ICs include programmed death‐1 (PD‐1), its primary ligand programmed death‐ligand1 (PD‐L1) and cytotoxic T‐lymphocyte‐associated antigen 4 (CTLA‐4) or CD152. Several additional inhibitory checkpoint targets such as Lymphocyte‐activation gene 3 (LAG‐3), T‐cell immunoglobulin (Ig) and mucin domain‐3 (TIM‐3) are on the spotlight as approved or emerging targets for cancer immunotherapy.^1^, ^2^ The Food and Drug Administration (FDA)‐approved ICI‐based oncological therapies and the percentage incidence of irAEs observed in response to treatment reported in clinical trials are summarized in Tables 1 and 2, respectively.

**TABLE 1:** FDA‐approved IC‐inhibiting antibody‐based cancer therapies.

Target	Generic name	Trade name	Developer	Approval year	Indications and use in combination therapy	Adverse event indication on FDA label boxed warning
PD‐1	Keytruda	Keytruda	Merck	2014	Unresectable or metastatic melanoma, adjuvant treatment following complete melanoma resection	
					Specific cases of non‐squamous NSCLC, metastatic squamous NSCLC; specific cases of metastatic, progressed NSCLC (single agent, adjuvant therapy or in combination with chemotherapy)	
					Metastatic, unresectable, progressed or recurrent HNSCC (single or in combination with chemotherapy)	
					Refractory or relapsed cHL, PMBCL (accelerated approval also for use as an additional regimen dosing)	
					Specific cases of locally advanced or metastatic UC (single or in combination with enfortumab vedotin); unresectable, metastatic or progressed cases of MSI‐H or dMMR solid tumours or locally advanced unresectable or metastatic MSI‐H or dMMR CRC; locally advanced unresectable or metastatic Her2‐positive gastric cancer or GEJ adenocarcinoma (in combination with trastuzumab and chemotherapy); locally advanced, unresectable or metastatic Her2‐negative gastric cancer or GEJ adenocarcinoma (in combination with chemotherapy)	
					Specific cases of locally advanced, unresectable or metastatic oesophageal or GEJ carcinoma (single or in combination with chemotherapy)	
					Specific cases of advanced, persistent, recurrent or metastatic cervical cancer (single agent or in combination with chemotherapy/ bevacuzimab); HCC; locally advanced, unresectable or metastatic BTC (in combination with chemotherapy); recurrent, locally advanced or metastatic MCC	
					Advanced RCC (single agent or in combination with chemotherapy), recurrent RCC (adjuvant therapy)	
					Specific cases of progressed non‐MSI‐H, pMMR, MSI‐H or dMMR, advanced endometrial cancer (single or in combination with chemotherapy)	
					Unresectable, metastatic or progressed (TMB‐H) [≥10 mutations/megabase (mut/Mb)] solid tumours (safety and efficacy with TMB‐H CNS cancers have not been established)	
					High‐risk, early‐stage TNBC; specific cases of locally recurrent unresectable or metastatic TNBC (single or in combination with chemotherapy)	
					Recurrent, metastatic or specific cases of locally advanced cSCC	
PD‐1	Nivolumab	Opdivo	Bristol‐Myers Squibb	2014	Unresectable or metastatic melanoma (single or in combination with ipilimumab); completely resected melanoma (adjuvant therapy)	
					Resectable NSCLC (as adjuvant therapy), specific metastatic cases or progressed NSCLC (single or in combination with chemotherapy/ ipilimumab)	
					Unresectable malignant pleural mesothelioma (in combination with ipilimumab)	
					Intermediate or advanced RCC (single or in combination with ipilimumab/ chemotherapy)	
					Progressed or relapsed cHL	
					Metastatic, recurrent or progressed SCCHN; high‐risk recurrent (adjuvant therapy) UC; locally advanced, metastatic, progressed UC	
					Progressed cases of MSI‐H or dMMR metastatic CRC (single or in combination with ipilimumab)	
					HCC (previously treated with chemotherapy, in combination with ipilimumab)	
					Residual pathologic disease in oesophageal or GEJ cancer; unresectable advanced recurrent or metastatic ESCC (in combination with chemotherapy or ipilimumab	
					Advanced or metastatic gastric, GEJ cancer and oesophageal adenocarcinoma (in combination with chemotherapy)	
PD‐1	Cemiplimab	Libtayo	Regeneron Pharmaceuticals	2018	Metastatic or locally advanced cSCC and BCC	
					Specific cases of metastatic, locally advanced, unresectable NSCLC (single or in combination with chemotherapy)	
PD‐1	Dostarlimab	Jemperli	Glaxo SmithKline	2021	dMMR‐recurrent or MSI‐H advanced EC (single agent, in combination with or following chemotherapy)	
					dMMR‐recurrent, advanced or progressed solid tumours	
PD‐1	Retifanlimab	Zynyz	Incyte	2023	Metastatic or recurrent locally advanced MCC	
PD‐1	Tislelizumab	Tevimbra	BeiGene	2024	Specific cases of unresectable or metastatic ESCC	
PD‐L1	Atezolizumab	Tecentriq^**$**^	Roche, Genentech	2016	Specific cases of NSCLC following chemotherapy (as adjuvant treatment), metastatic or progressed NSCLC, specific cases of metastatic non‐squamous NSCLC (in combination with bevacizumab and chemotherapy), ES‐SCLC (in combination with chemotherapy)	
					Unresectable or metastatic HCC (in combination with bevacizumab)	
					BRAF‐V600 mutation‐positive, unresectable or metastatic melanoma (in combination with chemotherapy)	
					Unresectable or metastatic ASPS	
PD‐L1	Avelumab	Bavencio^**##**^	Merck Serono, Pfizer	2017	Metastatic MCC, advanced RCC	
					Progressed, locally advanced or metastatic UC	
PD‐L1	Durvalumab	Imfinzi	AstraZeneca	2017	ES‐SCLC (in combination with chemotherapy)	
					Unresectable NSCLC (with concurrent chemotherapy and radiation therapy), metastatic or specific NSCLC cases (in combination with tremelimumab‐actl and chemotherapy), unresectable HCC (in combination with tremelimumab‐actl)	
					Locally advanced or metastatic BTC including cancer of bile ducts, cholangiocarcinoma (in combination with chemotherapy), gall bladder cancer	
CTLA‐4	Ipilimumab	Yervoy^**$$,**^ a	Bristol‐Myers Squibb	2011	Unresectable, metastatic melanoma (single agent or in combination with nivolumab) and special cases of cutaneous melanoma (adjuvant treatment)	
					Intermediate or poor‐risk advanced RCC; unresectable, advanced or metastatic ESCC (in combination with nivolumab)	
					Progressed MSI‐H or dMMR metastatic CRC, HCC following chemotherapy (in combination with nivolumab)	
					Unresectable malignant pleural mesothelioma (in combination with nivolumab)	
					Specific cases of metastatic or recurrent NSCLC (in combination with nivolumab)	
					Unresectable advanced or metastatic ESCC (in combination with nivolumab)	
CTLA‐4	Tremelimumab	Imjudo	AstraZeneca	2022	Unresectable HCC (in combination with durvalumab); Specific cases of metastatic NSCLC (in combination with durvalumab and chemotherapy)	
LAG‐3	Relatlimab/Nivolumab combination	Opdualag^**$#**^	Bristol‐ Myers Squibb	2022	Metastatic, unresectable melanoma	

*Note*: Indications and year of first approval for each antibody were accessed and compiled using the FDA drug database (https://www.accessdata.fda.gov/drugsatfda_docs/). Updated data for Tecentriq^**$**^, Bavencio^**##**^, Yervoy^**$$**^ and Opdualag^**$#**^ were accessed from ^**$**^ https://www.gene.com/download/pdf/tecentriq_prescribing.pdf, ^**##**^ https://www.drugs.com/pro/bavencio.html#S5, ^**$$**^ https://packageinserts.bms.com/pi/pi_yervoy.pdf, ^**$#**^ https://packageinserts.bms.com/pi/pi_opdualag.pdf, respectively.

Abbreviations: ASPS, alveolar soft part sarcoma; ALK, Anaplastic lymphoma kinase; BCC, basal cell carcinoma; BRAF‐V600, v‐Raf murine sarcoma viral oncogene homologue B amino acid, substitution at codon 600; BTC, biliary tract cancer; cHL, classical Hodgkin lymphoma; CNS, central nervous system; CRC, colorectal cancer; cSCC, cutaneous squamous cell carcinoma; CTLA‐4, cytotoxic T‐lymphocyte‐associated antigen 4; dMMR, mismatch repair‐deficient; EC, endometrial cancer; EGFR, epidermal growth factor receptor; ESCC, oesophageal squamous cell carcinoma; ES‐SCLC, extensive stage small cell lung cancer; GEJ, gastroesophageal junction; HCC, hepatocellular carcinoma; Her‐2, human epidermal growth factor receptor‐2; HNSCC, head and neck squamous cell cancer; IC, immune checkpoint; LAG‐3, Lymphocyte‐activation gene 3; MCC, Merkel cell carcinoma; MSI‐H, microsatellite instability‐high; NMIBC, non‐muscle invasive bladder cancer; NSCLC, non‐small cell lung cancer; PD‐1, programmed death‐1; PD‐L1, programmed death‐ligand1; PMBCL, primary mediastinal large B‐Cell lymphoma; pMMR, mismatch repair proficient; RCC, renal cell carcinoma; SCCHN, squamous cell carcinoma of the head and neck; TMB‐H, tumour mutational burden‐high; TNBC, triple‐negative breast cancer; UC, urothelial carcinoma.

^a^ FDA boxed warning for Yervoy was removed in June 2020 (https://www.accessdata.fda.gov/drugsatfda_docs/label/2020/125377s115lbl.pdf).

**TABLE 2:** IrAEs observed in clinical trials with approved IC‐inhibiting antibody‐based cancer therapeutics.

Indication	Keytruda	Opdivo	Libtayo	Jemperli	Zynyz	Tevimbra	Tecentriq^$^	Bavencio^##^	Imfinzi	Yervoy^$$,^ ^a^	Imjudo^b^	Opdualag^$#,^ ^c^
FDA label boxed warning												
CRS												
IM pneumonitis	3.4	3.1	2.6	2.3	3	3.8	3	1.1	2.4**	NA	1.3	3.7
IM colitis	1.7	2.9	2	1.3	1.6	0.9	1	1.5	2	12	6	7
IM hepatitis	0.7	1.8	2.4	0.5	3	1.7	1.8	1.1	2.8	4.1	7.5	6
IM Endocrinopathies (IM adrenal insufficiency)	0.8	1	0.5	1.2	0.7	0.3	0.4	0.6	0.5	4	1.5	4.2
IM hypophysitis	0.6	0.6	0.5	0.2	0.5	0.1	<0.1	0.1	<0.1	NA	1	2.5
IM thyroiditis	0.6	0.6	0.6	0.5	0.7	0.4	0.2	0.2	0.5	NA	1.5	2.8
Hyperthyroidism	3.4	2.7	3	2.3	6	0.6	0.8	0.4	2.1	NA	4.6	6
Hypothyroidism	8.0	8	7	8	10	7	4.9	5	8.3	NA	11	17
Type 1 diabetes mellitus	0.2	0.9	<0.1	0.2	0.2	NA	0.3	0.2	<0.1	NA	0.5	0.3
IM nephritis (with renal dysfunction)	0.3	1.2	0.7	0.5	1.6	0.4	<0.1	0.1	0.5	NA	1	2
IM dermatologic (including IM rash)	1.4	9	1.9	NA	8	1.2	0.6	6	1.8	15	4.9	9
IM encephalitis	<1	<1	<1	<1	<1	<1	<1	<1	<1	NA	<1	<1
Cardiac/vascular	<1	<1	<1	<1	<1	<1	<1	<1	<1	NA	<1	1.7^c^
Nervous system	<1	<1	<1	<1	<1	<1	<1	<1	<1	NA	<1	<1
Ocular	<1	<1	<1	<1	<1	<1	<1	<1	<1	NA	<1	<1
Gastrointestinal (including IM Pancreatitis)	<1	<1	<1	<1	<1	<1	<1	<1	<1	NA	2.3	<1
Musculoskeletal and connective tissue	<1	<1	<1	<1	<1	<1	<1	<1	<1	NA	<1	<1
Arthritis	1.5	<1	<1	<1	<1	<1	<1	<1	<1	NA	<1	<1
Endocrine: Hypoparathyroidism	<1	<1	<1	<1	<1	<1	<1	<1	<1	NA	<1	<1
IM Haematologic	<1	<1	<1	<1	<1	<1	<1	<1	<1	NA	<1	<1
Patient cohort size	2799	1994	1281	605	440	1972	2616	1854	1889 (*of 1414*)	511	388	355

*Note*: The table is compiled from data available on FDA drug database (https://www.accessdata.fda.gov/drugsatfda_docs/). Updated data for Tecentriq^**$**^, Bavencio^**##**^, Yervoy^**$$**^ and Opdualag^**$#**^ were accessed from ^**$**^ https://www.gene.com/download/pdf/tecentriq_prescribing.pdf, ^**##**^ https://www.drugs.com/pro/bavencio.html#S5, ^**$$**^ https://packageinserts.bms.com/pi/pi_yervoy.pdf; ^**$#**^ https://packageinserts.bms.com/pi/pi_opdualag.pdf, respectively. Numbers represent percentage (%) of incidence of specific irAE (including fatal, Grade 5, 4, 3 or 2 adverse reactions) observed in patients receiving the lowest dose of ICI‐cancer therapeutic administered as a single agent. Severe or fatal cases have been reported for some of the adverse reactions with an incidence of <1%.

Abbreviations: CRC, colorectal carcinoma; CRS, Cytokine release syndrome; IC, immune checkpoint; IM, immune‐mediated; irAE, immune‐related adverse event; NA, not available; RCC, renal cell carcinoma.

** Percentage reported is based on a patient cohort size of 1414.

^a^ FDA boxed warning for Yervoy was removed in June 2020 (https://www.accessdata.fda.gov/drugsatfda_docs/label/2020/125377s115lbl.pdf).

^b^ Numbers represented for Imjudo are irAE incidence (%) in combination with durvalumab, since the antibody is used as a combination therapy with durvalumab (as shown in Table 1).

^c^ Myocarditis leading to Opdualag discontinuation, systemic corticosteroid administration resolved myocarditis in all these patients. The patient cohort size used for determination of irAE (%) is shown in the last row (highlighted). **Percentage reported is based on a patient cohort size of 1414.

The first ICI Ab approved for clinical use was the anti‐CTLA‐4 Ab, ipilimumab. CTLA‐4 is an activation‐induced Ig‐superfamily receptor, expressed by conventional T cells. Due to homology with the primary T‐cell co‐stimulatory protein, CD28, both CTLA‐4 and CD28 compete for CD80/CD86 binding on antigen‐presenting cells (APCs). CTLA‐4 binds CD80/CD86 with greater affinity and avidity than CD28 and contributes to negative modulation of T‐cell activity in the early stages of peripheral T‐cell priming, by outcompeting CD28 at the immunological synapse.^3^ CTLA‐4 can extract ligands CD80/CD86 from the APC surface, thereby downmodulating T‐cell activation.^4^ Thus CTLA‐4 blockade leads to effective T‐cell priming at lower activation thresholds due to enhanced availability of CD80/CD86 co‐stimulation. In addition to co‐stimulation inhibition, CTLA‐4 blockade also attenuates regulatory T‐cell (T_reg_) recruitment to the tumour site, leading to improved infiltration of tumour‐infiltrating lymphocytes (TILs).^5^ A direct, undesirable outcome of increased activation is the incremented likelihood of self‐directed immunological response. CTLA‐4 inhibition‐derived toxicities manifest from heightened co‐stimulatory signal, leading to CD28 phosphorylation via downstream Ak strain transforming (AKT) kinase and protein kinase C‐θ (PKC‐θ) activation, that in turn leads to enhanced T‐cell‐mediated interleukin (IL)‐2 production, and proliferation orchestrated by mammalian target of rapamycin (mTOR) and nuclear factor‐ κB, NF‐κB.

Today, the majority of the approved IC drugs target the PD‐1/PD‐L1 axis and there is an emerging move towards combination strategies.^6^, ^7^, ^8^ PD‐1 belongs to the CD28 family of immunoregulatory receptors and binds the distinct ligand, PD‐L1 and, to a lesser extent, PD‐L2, helping maintain peripheral tolerance.^9^ Most circulating T cells do not express PD‐1 until direct T‐cell receptor (TCR) activation; MHC‐associated antigen; or cytokine stimulation, following which elevated PD‐1 expression at the immunological synapse functions for direct and indirect immune regulation.^10^ Tyrosine phosphorylation of intracellular immunoreceptor tyrosine‐based inhibitory motifs (ITIMs) and immunoreceptor tyrosine‐based switch motifs (ITSMs) follows ligand binding. Consequent recruitment of src homology region 2 domain‐containing phosphatase‐1 and 2 (SHP1/SHP2) dampens TCR signalling due to downmodulation and reduced zeta‐chain‐associated protein kinase 70 (ZAP70) phosphorylation/activation.^11^ Binding of PD‐1 causes upregulation of E3‐ubiquitin ligases, CBL‐b and c‐CBL and consequent downmodulation of unengaged TCRs and/or TCR retention during stages of recycling.^12^ Toxicities arising from anti‐PD‐1 or anti‐PD‐L1 therapy are therefore propagated by unchecked and physiologically aberrant levels of TCR‐mediated signalling. The overall patient response to ICI is heterogeneous with most cancer types showing response rates ranging from 15% to 40%, based on the tumour type,^13^ while CTLA‐4 therapy delivers an objective response rate of 20% in metastatic melanoma patients.^14^ PD‐1 and PD‐L1 have been tested more widely on various tumours. A comprehensive meta‐analysis assessing the benefit of PD‐1/PD‐L1 therapy in cancer patients has shown the response rates typically fall in the range from 10% to 30% in various carcinogen‐induced solid tumours including those affecting the liver, bladder and kidneys.^15^ Relatively high response rates ranging from 40% to 62% were observed in cancers with higher immunogenicity, including Hodgkin lymphoma, Merkel cell cancer (MCC) and cutaneous cancer (CC), microsatellite unstable and/or mismatch repair‐deficient cancers including strong PD‐L1 expressing non‐small‐cell lung cancer (NSCLC).^15^, ^16^ These outcomes could also be based on the clinical status of the patient, the therapeutic pathway being targeted for treatment, clinical trial protocol and design for evaluating outcomes. A small proportion of patients on PD‐1 therapy may develop hyper‐progressive disease due to T_reg_‐mediated suppression of anti‐tumour immunity.^17^ Unfortunately, many patients receiving ICI therapy derive marginal or no benefit at the expense of suffering side effects. It is also important to recognize that the incidence, severity, and spectrum of organ systems affected by irAEs are very broad. Toxicities maybe observed in specific or multiple organs and can vary in incidence and severity. Some studies have reported a higher incidence of PD‐1 blockade‐related pneumonitis in NSCLC and renal cell carcinoma (RCC) than in melanoma,^18^ whereas GI, arthritis, myalgia and skin irAEs were more frequently observed in melanoma patients.^19^ Dyspnoea was more prevalent in RCC patients.^19^ Incidence of pneumonitis was found higher following ICI therapy targeting the PD‐1/PD‐L1 axis either as monotherapy or in combination, as opposed to patients receiving anti‐CTLA4 therapy.^20^ While in some cases, the affected organ maybe the same as is being targeted for therapy (as in the case of skin‐related AE such as rash occurring in melanoma patients); the relationship between a particular tumour type and the observed irAE is unclear in cases such as incidence of dyspnoea in RCC patients. The site of metastasis, existing comorbidities, the treatment modality and prior therapies may additionally impact the incidence of irAEs in particular tumour types.^19^ Despite the above, clinically responsive patients are found to demonstrate good responses and cases of long‐term remissions have been reported in the responding patient cohort highlighting the value of the treatment. Due to the positive correlation between IC target expression and clinical success, stratification of patients for ICI therapy is currently a subject of great interest, though patient response rate can vary despite high marker expression.^21^ Predictive biomarkers for clinical response could help maximize the clinical utility of ICI therapy.^22^, ^23^ Tumour mutational burden (TMB) has been shown as a significant factor determining therapeutic success.^24^, ^25^ The positive correlation of higher TMB, with enhanced neoantigen production and consequently broader anti‐tumour T‐cell response and improved clinical outcomes was first recognized in melanoma patients treated with ipilimumab or tremelimumab.^26^ Several studies report improved ICI efficacy in cancers with high TMB^27^, ^28^, ^29^ and a positive correlation with irAEs has been reported across multiple tumour types following ICI therapy.^30^, ^31^ Interestingly, evidence from immune‐privileged tumours such as gliomas or glioblastomas reveals a correlation of low TMB with improved survival responses.^32^, ^33^ Thus, while the adaptation of a specified TMB scoring system may be constrained for broader applicability, harmonization of TMB measurements when considering treatment decisions for patients will be important for adaptation in clinical settings.^27^, ^28^, ^29^ There is contradictory evidence on association of human leukocyte antigen (HLA) signatures and clinical outcomes. Studies have shown the positive association of the HLA‐B44 class I supertype, influenced by HLA‐B*18:01, HLA‐B*44:02, HLA‐B*44:03, HLA‐B*44:05 and HLA‐B*50:01, on improved survival of melanoma patients treated with PD‐1 or CTLA‐4 targeting Abs; while HLA‐B62 association driven by the HLA‐B*15:01 allele was found to associate with poor survival outcomes.^34^ Melanoma patients heterozygous for all HLA‐class‐I alleles revealed broader TCR clonal diversity during anti‐PD1 therapy, driven by the increased diversity of displayed tumour and neoantigen peptides.^34^ Interestingly the positive influence of HLA‐B44 on survival was not observed in NSCLC patients despite a similar mutational burden status to melanoma.^35^ These studies suggest that comprehensive analysis of HLA association and mutational patterns can provide valuable insights and inform selection criteria for ICI therapy initiation.^36^ However, this could be restricted to specific patient subsets displaying specific mutations and further studies are necessary for deriving associations in cancers with lower mutational burdens such as neoplasms.^37^ While a lack of general association is observed between HLA and overall irAE development upon ICI therapy in metastatic melanoma and NSCLC patients, the association between HLA‐class‐II alleles such as HLA‐DRB1*11:01 and HLA‐DQB1*03:01 that are linked to autoimmune diseases and the propensity for developing organ‐specific irAEs such as pruritis, colitis and flare‐up of existing autoimmune conditions upon ICI therapy has been reported.^38^ The degree of HLA‐A*03 heterozygosity was reported to have an additive impact and is proposed as a potential predictive biomarker for poor response to ICI therapy in patients with particular cancers including melanoma, NSCLC, bladder cancer, glioma and RCC.^39^ HLA homozygosity is reported to be associated with inferior outcomes in NSCLC patients undergoing ICI monotherapy.^40^ Patients developing endocrine irAEs were found to display exclusive HLA‐class‐II alleles, such as DRB4, also associated with improved overall survival in metastatic NSCLC patients receiving ICI therapy.^41^ Recent research has demonstrated the contributory role of the microbiome in supporting and overcoming resistance to ICI therapy.^42^, ^43^ Evidence for the influence of gut microbiome composition is supported by the observed poor efficiency of ICI therapy in germ‐free preclinical models and patients undergoing antibiotic treatment prior to ICI therapy.^44^, ^45^ Studies have reported the influence of microbiome features on response to ICI therapy and the development of irAE such as colitis.^46^ The microbiota profile in healthy individuals versus cancer patients shows inherent heterogeneity, adding another level of complexity to the impact of microbiome on clinical benefit.^47^ Faecal microbiota transplantation (FMT) from immune or ICI therapy‐responsive patients to refractory melanoma patients is reported to improve response to ICI therapy; and clinical trials using FMT, probiotics or prebiotics to improve efficacy in patients with resistant or recurrent tumours are underway for several cancer indications including non‐responder melanoma, gastrointestinal (GI), renal cell or head and neck carcinoma, advanced lung cancer, NSCLC, colorectal, liver, prostate cancer and patients with solid tumours treated with dual ICIs.^42^, ^48^, ^49^ Significant differences in the association of microbial abundance and diversity of certain microbial species such as *Ruminococcaceae* in melanoma^50^ and *Akkermansia* and *Enterococcus* in patients with advanced NSCLC or urothelial carcinoma patients have been demonstrated in the patient gut microbiome of responders versus non‐responders to ICI therapy.^45^, ^50^ Molecular mimicry between microbial and tumour antigens is another mechanism contributing to the antigen‐specific arm of responses and recent studies demonstrate the ability of intra‐tumoral bacteria to directly modulate anti‐tumour immune responses.^51^ In addition to tumour antigen‐directed cytotoxic T‐cell (CTL) responses, systemic effects mediated by T‐helper (Th)‐1 and antigen‐independent mucosal‐ and toll‐like receptor (TLR)‐7 and 9‐mediated innate immune responses result in Th‐2 and NK‐cell activation, contributing to tumour directed immune response. Leveraging the modulatory role of microbiota may however not be uniformly applicable to all patient subsets and combinatorial approaches to limit immune‐mediated (IM) toxicities and/or improve efficacy, are likely to be required.^52^, ^53^ Identifying interactions and the specific microbial populations influencing anti‐tumour responses and irAE development is an interesting research focus.^54^, ^55^ Efforts on standardization for harmonized and detailed profiling of the gut and tumour microbiome signatures will support the comprehensive understanding of cancer immunotherapy responses and associated toxicities and enable integration of the microbiome‐based therapies as an adjunct for cancer treatment. Overall, response to ICI can exhibit significant variability, is multivariant and determined by an interplay of several complex pathways at and distant from the tumour site. These could include predefined germline genetic characteristics leading to varying levels of HLA‐associated molecular diversity, microbiome composition and tumour intrinsic features including mutational quality that could shape an anti‐tumour response by influencing TCR clonal diversity and contributing to improved clinical benefit.^56^

Since ICI therapies channel their application via T‐cell action and not directly via tumour cells, the toxicities observed vary from those commonly noted with chemotherapy. Almost from 70% to 90% of patients develop IM adverse reactions (imAR) and from 5% to 10% of patients develop severe Grade 3 and Grade 4 toxicities stemming from severe systemic inflammation leading to treatment suspension.^57^, ^58^ Some irAE observed following ICI treatment have been observed in preclinical models.^59^ During preclinical development of tremelimumab, both skin rash and persistent loose stools with weight loss were noted in toxicology studies in cynomolgus monkeys correlating with the dose‐limiting toxicities seen in clinical trials.^60^ Experiments in human CTLA‐4 knock‐in mice, showed that severe irAE induction with ipilimumab, especially when combined with an anti‐PD‐1 Ab.^61^ Perinatal mice‐human CTLA‐4 knock‐in mice display rapid tumour regression in response to ipilimumab, coupled with severe organ inflammation in heart, lung, liver and kidney. Transmural inflammation in the colon, a unique pathological feature of Crohn's disease, was also observed in these mice upon anti‐PD‐1 and ipilimumab treatment. Systemic T‐cell activation and reduced T_reg_ to autoreactive effector T‐cell ratios were correlated with irAE occurrence.^61^ Interestingly, studies with anti‐CTLA‐4 therapy suggest a lack of correlation between toxicity and drug efficacy, while more recent studies with PD‐1 targeting solid tumour treatment have observed irAE incidence correlating with improved response rate and survival compared with patients lacking toxicity.^62^, ^63^ irAE onset could manifest immediately following infusion or several months following treatment initiation.^64^ Most common side effects include fatigue, rash, pruritus, diarrhoea, vitiligo, hormone, haematological imbalances, rheumatological disorders, and pneumonitis. Systemic pro‐inflammatory cytokine induction could initiate irAEs and more severe responses can occur in some patients due to unimpeded T‐cell activation. Severe events could lead to colitis, hepatitis, renal injury, neurological, and cardiac dysfunction. Though dermatologic events such as rash, pruritus, and vitiligo are usually the first to manifest, self‐targeting T cells can affect a wide range of organ systems, including GI tract, causing diarrhoea and colitis; endocrine glands, leading to hypo−/hyperthyroidism; and lung and liver inflammation, causing pneumonitis and hepatitis; respectively.^65^, ^66^ Expansion of autoantigen, α‐myosin‐reactive clones was found to associate with ICI‐induced myocarditis.^67^ Differentiation and expansion of gut mucosal Th‐17 cells may play an important role in the pathogenesis of GI‐related irAEs^68^ and consequently administration of gut microbiota‐based biopharmaceuticals and modulating the pro‐inflammatory activities of Th‐17 cells via monoclonal Ab (mAb)‐based IL‐17A or receptor targeting hold promise.^69^ Percentage incidence of the spectrum of irAE observed following FDA‐approved ICI therapy is summarized in Table 2.

Due to inherent distinctions in the mechanistic action of anti‐CTLA‐4 therapy, this modality, in general, associated with frequent and more severe irAE, in comparison with PD‐1/PD‐L1‐directed therapy.^66^ Genetic ablation of *Ctla‐4* in mice is reported to cause premature lethality due to uncontrolled lymphoproliferation and activated T‐cell infiltration leading to multiorgan autoimmunity.^70^ In contrast, *Pd‐1* or *Pd‐l1* deficient mice display relatively slower onset of a spectrum of lupus‐like arthritis, glomerulonephritis and cardiac pathology associated with high levels of circulating autoantibodies.^71^, ^72^ Interestingly, these phenotypes broadly correlate with the higher incidence of dose‐dependent toxicities associated with CTLA‐4 inhibition (38.6% and 57.9% of metastatic melanoma patients receiving higher doses of ipilimumab experienced high‐grade toxicities^73^), in comparison with PD‐1/ PD‐L1 blockade reported to cause high‐grade adverse events from 10% to 15% of patients, over a range of doses,^74^ indicating the improved tolerance of PD‐1/PD‐L1 inhibitors in cancer patients. FDA boxed or black box warning is the strongest caution issued for a specific therapy to alert healthcare providers and patients of significant and potentially serious side effects associated with a medicine or any restrictions on its use. The warning box associated with Yervoy indicating imARs was recently removed (https://www.accessdata.fda.gov/drugsatfda_docs/label/2020/125377s115lbl.pdf) and consolidated within Section 5 of the product labelling due to FDA recognition of a better understanding of identification and management of associated irAE since the initial approval in 2015. Not surprisingly, combined ICI therapy significantly increments the incidence of high‐grade irAEs and autoimmune toxicities leading to treatment discontinuation for example in >20% of advanced RCC patients in the combined treatment group.^75^ Notably, clinical factors related to CRS are not well described and may often not be reported in the FDA label boxed warning.^76^ Overall differences in irAE localization and severity are observed though the underlying mechanisms are not completely understood.^19^ Association of vitiligo used historically to predict successful clinical outcomes in melanoma patients and has been correlated with successful ICI outcome in some studies.^77^ While most irAEs can be clinically managed using immunosuppressive drugs with close monitoring of symptoms and blood parameters, discontinuation of treatment may be necessary in patients presenting more severe, uncontrolled responses.^78^, ^79^ Predicting, understanding, and timely management of toxicities are therefore crucial components in the pathway to derive therapeutic benefit.^13^ The development of optimized therapeutic strategies is a current research focus and the identification of predictive biomarkers will enable a deeper understanding of patient response.

In an attempt to circumnavigate the need for immunosuppressive drugs, research has focussed on targeted delivery systems as innovative approaches for tumour‐selective delivery, to limit on‐target/off‐tumour side effects to produce safer and more effective immunotherapeutic modalities. The potential of recombinant adeno‐associated (rAAV) and oncolytic vaccinia virus to selectively target PD‐1 and CTLA‐4‐based therapy to the tumour site shows promise in preclinical evaluations.^80^, ^81^ Small interfering (si) ribonucleic acids (RNAs), micro RNAs (miRNAs), peptides, and small molecules incorporated as nano systems offer hope for improving the efficacy of ICI in cancer treatment.^82^ Nanoparticles (NPs) can be targeted to tumours by coating with ligands or antibodies that recognize and bind to receptors or antigens which are over‐expressed or selectively expressed on tumour cells, or targeted to characteristic facets of the tumour microenvironment (TME) such as pH, temperature or enzyme concentration.^83^ PD‐L1‐targeting siRNA/ miRNA loaded NPs could achieve targeted gene silencing within mouse colon carcinoma and human epithelial ovarian cancer cells.^84^, ^85^, ^86^ The clinical potential of siRNA‐mediated PD‐L1 knockdown was demonstrated in a preclinical humanized mouse model of pancreatic cancer (known to be resistant to ICI therapy).^87^ PD‐L1 siRNA co‐delivered with the oncogenic transcription factor, signal transducer and activator of transcription‐3 (STAT3) siRNA as specialized nanoparticles were found to constrain tumour growth in murine melanoma and breast cancer models^88^; IL‐2 siRNA in activated T‐cell/ lung cancer cell coculture models^89^ and co‐suppression with PD‐1 siRNA demonstrated potent tumour repression in colon cancer mouse xenograft models.^90^ PD‐L1‐targeting peptides and small molecules (such as BMS‐202) loaded into NPs have also shown reductions in tumour‐bearing mouse models.^82^ Dual pH‐sensitive nanoparticle design‐based combination therapy of anti‐PD‐1 coupled with NF‐κB inhibitor, curcumin, was found to improve anti‐tumour response generation, with reduced side effects.^91^ Camelid‐derived, nanobodies targeting PD‐L1 and CTLA‐4, expressed in probiotic *E. coli*, provided a means of specific delivery achieving selective intra‐tumoural bacterial colonization and therapeutic efficacy in murine tumour models.^92^ These approaches highlight few of the inventive means investigating more targeted drug delivery systems. These drugs may have the potential to benefit many types of cancer, but their most profound advantages may lie in reducing off‐tumour effects.

## 3 BsAbs AND BISPECIFIC T‐CELL ENGAGERS (BiTEs)

BsAbs are designed with two different antigen‐binding domains, which could recognize either a different epitope of the same antigen or two separate antigens. Depending on the design, a BsAb could recognize a tumour‐associated antigen (TAA) such as CD19, CD20, CD123, B‐cell maturation antigen (BCMA) or human epidermal growth factor receptor 2 (HER2), on one arm and function as a T‐cell engager via CD3 or co‐stimulatory molecules on the other arm.^93^ Alternatively, BsAbs may engage other immune cell receptors, activating a directed anti‐tumour response.^94^ BsAbs have a myriad of different formats that can vary with respect to molecular weight, antigen‐binding site valency, receptor affinity, spatial relationship between moieties, Fc‐mediated effector functions^95^, ^96^, ^97^ etc. Early‐phase clinical trials for solid tumour indications have shown promise with BsAbs simultaneously targeting different immune receptors including tumour intrinsic IC‐protein such as PD‐1/L1, CTLA‐4 and LAG3.^98^, ^99^ Based on the natural killer (NK) or T‐cell binding potential, BsAbs can be classified as NK‐cell engagers (NKCEs) or T‐cell engagers (BiTEs).^100^, ^101^, ^102^ NKCEs including bispecific or trispecific killer‐cell engagers (BiKEs or TriKEs) are a novel class of Ab‐based therapeutics currently in development.^103^ BiTEs are a specific BsAb subtype designed to engage T cells and cancer cells and are usually composed of two Ab single‐chain variable fragments (scFv) connected by a short linker. Approved BsAbs are summarized in Table 3.

**TABLE 3:** Approved BsAbs/BiTEs for cancer treatment.

Target	Generic name	Trade name	Developer	Approval year	Indications and use in combination therapy	Adverse event indication on FDA label boxed warning
CD19 × CD3	Blinatumomab	Blincyto	Amgen	2014	Relapsed or refractory CD19 positive B‐cell precursor ALL	CRS
					Specific MRD positive, CD19 positive B‐cell precursor ALL cases in first or second complete remission with ≥0.1% MRD	Neurological toxicities including ICANS
EGFR × MET	Amivantamab‐vmjw	Rybrevant	Janssen	2021	Locally advanced or metastatic NSCLC with specific EGFR exon 20 mutations (single or in combination with chemotherapy)	
gp100 × CD3	Tebentafusp‐tebn	Kimmtrak	Immunocore	2022	Unresectable or metastatic HLA‐A*02:01‐positive, uveal melanoma	CRS
BCMA × CD3	Teclistamab‐cqyv	Tecvayli^a^	Janssen	2022	Specific cases of relapsed or refractory multiple myeloma	CRS
						Neurological toxicity including ICANS
CD20 × CD3	Mosunetuzumab‐axgb	Lunsumio	Roche/Genentech	2022	Specific cases of relapsed or refractory follicular lymphoma	CRS
PD‐1 × CTLA‐4	Cadonilimab^b^		Akeso Inc.	2022	Specific cases of relapsed, progressed or metastatic cervical cancer	N/A
CD20 × CD3	Glofitamab‐gxbm	Columvi	Roche	2023	Specific cases of relapsed or refractory DLBCL, NOS	CRS
					Specific cases of relapsed or refractory LBCL arising from follicular lymphoma	
CD20 × CD3	Epcoritamab‐bysp	Epkinly/Tepkinly	Genmab	2023	Specific cases of relapsed or refractory DLBCL, NOS including DLBCL arising from indolent lymphoma	CRS
					Specific cases of high‐grade BCL	ICANS
GPRC5D × CD3	Talquetamab‐tgvs	Talvey^a^	Janssen	2023	Specific cases of relapsed or refractory multiple myeloma	CRS
						Neurological toxicity including ICANS

*Note*: Indications and year of first approval for each antibody were accessed and compiled using the FDA drug database (https://www.accessdata.fda.gov/drugsatfda_docs/). Updated information for tecvayli was accessed from (https://www.janssenlabels.com/package‐insert/product‐monograph/prescribing‐information/TECVAYLI‐pi.pdf).

Abbreviations: ALL, Acute lymphoblastic leukaemia; BCL, B‐cell lymphoma; BCMA, B‐cell maturation antigen; BiTEs, bispecific T‐cell engager; BsAbs, bispecific antibodies; CRS, cytokine release syndrome; CTLA‐4, cytotoxic T‐lymphocyte‐associated antigen 4; DLBCL, diffuse large B‐ cell lymphoma; EGFR, epidermal growth factor receptor; gp‐100, glycoprotein 100; GPRC5D, G protein‐coupled receptor class C group 5 member D; HLA, human leukocyte antigen; ICANS, immune effector cell associated neurotoxicity syndrome; LBCL, large B‐ cell lymphoma; MET, mesenchymal–epithelial transition factor; MRD, minimal residue disease; NOS, not otherwise specified; NSCLC, non‐small cell lung cancer; PD‐1, programmed death‐1; PD‐L1, programmed death‐ligand1; REMS, risk evaluation and mitigation strategy.

^a^ Available only through a restricted program called the Tecvayli and Talvey REMS.

^b^ Approved in China.

Most approved BsAbs drive efficient tumour killing via CD3 targeting and subsequent immune synapse formation at the T‐cell‐TAA interface. The downside of a CD3‐directed approach is the initiation of a strong polytypic T‐cell response involving multiple subsets. CD4^+^ Th cell activation‐derived cytokines could contribute to increased CRS risk, while polyclonal T‐cell activation, and redirection of naïve, exhausted T cells, or T_reg_ could also drive ‘on‐target/off‐tumour’ T‐cell redirection to normal tissues bearing TAA leading to unwanted side effects. Elevated IL‐10, IL‐6, and interferon‐gamma (IFN‐γ) levels were reported in patients receiving blinatumomab therapy. Given that IL‐6 and IL‐10 elevation is not explained entirely by CTL activation, haemophagocytic lympho‐histiocytosis or macrophage activation syndrome could be implicated in CRS pathophysiology following blinatumomab therapy.^104^, ^105^ For CD19‐binding Abs such as blinatumomab, the burden of target cells is increased since both malignant and normal B‐cells express the CD19‐target antigen. This increased target burden may widen the spectrum and severity of observed irAEs. Severe CRS is reported more frequently in patients with high disease burden. Glofitamab, a recently approved CD20 x CD3 BsAb, with a molecular configuration allowing for bivalent binding on CD20 and monovalent binding ratio (2:1) shows superior potency to other 1:1 configuration BsAb.^106^ Obinutuzumab (humanized anti‐CD20 mAb) pre‐treatment and step‐up dosing approach were found to be effective in mitigating CRS risk in patients with refractory or relapsed non‐Hodgkin lymphoma administered glofitamab.^107^ Systemic CRS is expected to be less frequent in the context of solid tumours, where target cells are less numerous. The aetiology of neurotoxicity associated with CD3‐BsAbs is also possibly related to elevated cytokine levels, though a complete understanding of the underlying mechanisms has not been fully achieved. Talvey and Tecvayli, approved for specific relapsed or refractory multiple myeloma cases, are available only through the risk evaluation and mitigation strategy (REMS), due to the reported risk of CRS and neurological toxicity including immune effector cell associated neurotoxicity syndrome (ICANS), reported in clinical trials, highlighted in the FDA boxed warning (Table 3). Recent research sheds light on critical aspects of CRS and provides insights for clinical management of CRS following BsAb treatment.^108^ Using single‐cell RNA and bulk RNA sequencing, the researchers identified T‐cells as initial drivers of cytokine and chemokine release, activating a myeloid‐cell‐coordinated downstream cascade of events. These studies identify the significant role of endothelial cells and neutrophil‐derived mediators as key contributors to inflammatory response elicited by BsAb such as blinatumomab.^108^, ^109^ Preclinical models demonstrate the role of monocytes and macrophages to CRS development.^110^, ^111^, ^112^ Studies reporting dissociation of the systemic CR potential and T‐cell cytotoxicity promoted by CD3‐targeting BsAbs provide insights on approaches to clinically manage CRS by moderating TNF‐α and IL‐6^109^, ^113^ and improve BsAb design. Due to a greater incidence of BsAb treatment‐associated‐irAEs, intense efforts are currently focussed on assessing preclinical and translational safety of CD3‐targeting BsAbs.^114^ Alternate strategies for CD8^+^ and/or memory T‐cell redirection could not only improve tumour cell killing responses but also limit irAEs.^115^, ^116^ Targeting pathways other than T‐cell‐engaging or non‐bCD3‐directed pathways may be another approach to improve tolerability in patients by reducing pan T‐cell activation and associated CRS risk, as demonstrated in clinical trials leading to the approval of amivantamab. Epidermal growth factor receptor (EGFR) × mesenchymal–epithelial transition factor (MET)‐targeting BsAb amivantamab, functions via blocking ligand‐induced activation and leads to internalization and degradation of Ab‐bound receptors, thereby inhibiting tumour cell proliferation. Amivantamab treatment leads to monocytic Fc‐receptor‐mediated trogocytosis as a crucial inducer of target receptor downmodulation.^117^ In development of future BiTEs, improved design of the CD3‐targeting arm can additionally minimize cytokine release (CR) potential while maintaining tumour‐targeted cytotoxicity.^118^ Combining oncolytic therapy could be another approach to circumvent BiTE‐associated off‐target toxicities and improve specificity. Oncolytic viruses with specific tumour tropism ‘armed’ with BiTE sequence can induce selective BiTE expression at the tumour site and have shown promise in solid tumour models in vivo.^119^ Approaches evaluating engineered oncolytic virus enadenotucirev (EnAd), encoding specific BiTEs demonstrate tumour‐specific expression and killing.^120^ Although promising, these technologies pose a cumulative risk of AEs induced by the oncolytic virus, and therefore the immune response to virus‐infected cells, tumour cell behaviour, and BiTE activity will need extensive preclinical validation. Consolidating efforts on identifying and defining suitable thresholds for immune activation required to elicit an anti‐tumour response versus uncontrolled CR, though challenging, will be central to steering away from treatment‐associated toxicity profiles towards improved therapeutic efficacy.

## 4 Ab‐DRUG‐CONJUGATES

Ab‐drug‐conjugates use selective binding properties of Abs for targeted delivery of cytotoxic agents to tumour cells, a concept of ‘magic bullets’ first conceived by Paul Ehrlich in the early 1900s. While, in principle, the strategy offers an attractive solution for directed delivery and increasing therapeutic index of oncological therapies, several factors contribute to clinical success. All three ADC constituents namely the Ab, conjugated drug/payload, and linker, are crucial to an effective ADC design. Notwithstanding the above, choice of the antigenic target based on selective expression in tumour versus non‐tumour tissues, is perhaps the most critical for the development of an effective ADC. Conjugation of cytotoxic agents to larger and intrinsically hydrophilic Ab molecules could physically limit drug entry into non‐tumour cells lacking target antigen. In addition, since the ADC pharmacokinetic features are determined by the targeting Ab, systemic side effects could in theory be minimized. Approved ADCs are summarized in Table 4. Despite ADCs being designed for directed delivery, AE incidence is high. Haemotoxicity, hepatotoxicity and GI reactions may occur due to premature systemic release of cytotoxic payloads in the bloodstream and immune responses induced by Abs to ADCs can additionally cause secondary damage. The dose at which off‐target toxicity is observed in treatment regimens is dependent on the ADC design. Studies have compared AE occurrence, differences in Ab target and payloads, following ADC therapy.^121^

**TABLE 4:** Approved ADCs for cancer treatment.

Target	Generic name	Trade name	Developer	Approval year	Indications and use in combination therapy	Adverse event indication on FDA label boxed warning
CD30	Brentuximab‐vedotin	Adcetris	Seattle Genetics/Takeda	2011	Stage III or IV cHL, high‐risk cHL (in combination with chemotherapy), relapsed or progressed cHL cases	JC virus infection resulting in PML
					CD30 expressing PTCL, including angioimmunoblastic TCL (in combination with chemotherapy)	
					Relapsed pcALCL or CD30‐expressing MF	
					Progressed, relapsed or previously untreated sALCL	
CD22	Inotuzumab‐ozogamicin	Besponsa	Pfizer/Wyeth	2017	Relapsed or refractory CD22‐positive B‐cell precursor ALL	Hepatotoxicity, including fatal and life‐threatening VOD
						Increased risk of post‐HSCT non‐relapse mortality
CD33	Gemtuzumab‐ozogamicin	Mylotarg	Pfizer/Wyeth	2017	Newly diagnosed CD33‐positive AML, except acute APL	Hepatotoxicity, including fatal and life‐threatening veno‐occlusive disease (VOD)
CD79b	Polatuzumab‐vedotin	Polivy	Roche/Genentech	2019	Untreated, relapsed or refractory DLBCL, NOS or not otherwise specified or HGBL	
HER2	Trastuzumab‐emtansine	Kadcyla	Roche/Genentech	2013	Specific cases of metastatic, progressed or recurrent HER2‐positive breast cancer; HER2‐positive early breast cancer with residual invasive disease (adjuvant treatment)	Hepatotoxicity, fatal liver failure
						Reduction in LVEF
						Embryo‐foetal toxicity during pregnancy
HER2	Trastuzumab deruxtecan	Enhertu	AstraZeneca/Daiichi Sankyo	2019	Specific cases of unresectable, metastatic or recurrent HER2‐positive breast cancer	LD and pneumonitis
					Progressed, recurrent, unresectable or metastatic HER2‐low breast cancer	Embryo‐foetal toxicity during pregnancy
					Locally advanced or metastatic HER2‐positive gastric or GEJ adenocarcinoma (following trastuzumab treatment)	
					Unresectable or metastatic NSCLC with activating HER2 mutations	
HER2	Disitamab vedotin	Aidixi^a^	Remegen	2021	Urothelial and gastric cancer	N/A
Nectin‐4	Enfortumab vedotin	Padcev	Astellas/Seagen Genetics	2019	Specific cases of locally advanced or metastatic UC (single or in combination with pembrolizumab)	Severe and fatal cutaneous adverse Grade 3 or higher reactions, including SJS and TEN
EGFR	Cetuximab saratolacan	Akalux^b^	Aspyrian Therapeutics/Rakuten medical	2020	Unresectable locally advanced or recurrent head and neck cancer	N/A
Trop‐2	Sacituzumab govitecan	Trodelvy	Immunomedics/Gilead Sciences	2020	Specific cases of unresectable locally advanced or metastatic TNBC; unresectable locally advanced or metastatic HR‐positive, HER2‐negative breast cancer	Severe or life‐threatening neutropenia
					Specific cases of unresectable locally advanced or metastatic UC	Severe diarrhoea
Tissue factor	Tisotumab vedotin‐tftv	Tivdak	Seagen Inc.	2021	Recurrent, metastatic or progressed cervical cancer	Ocular toxicity including changes in corneal epithelium and conjunctiva leading to vision changes, severe loss of vision and corneal ulceration
CD19	Loncastuximab tesirine‐lpyl	Zynlonta	ADC Therapeutics	2021	Relapsed or refractory LBCL	
					DLBCL, NOS; DLBCL arising from low grade lymphoma and high‐grade B‐cell lymphoma	
Folate receptor, FRα	Mirvetuximab soravtansine	Elahere	ImmunoGen (Abbvie)	2022	Select cases of FRα‐ positive, platinum‐resistant epithelial ovarian, fallopian tube or primary peritoneal cancer	Severe ocular toxicity including visual impairment, keratopathy, dry eye, photophobia, eye pain and uveitis

*Note*: Indications and year of first approval for each antibody were accessed and compiled using the FDA drug database (https://www.accessdata.fda.gov/drugsatfda_docs/). Updated information for Besponsa and Mylotarg was accessed from (https://labeling.pfizer.com/ShowLabeling.aspx?id=9503&format=PDF and https://labeling.pfizer.com/ShowLabeling.aspx?id=13965), respectively. ^a^Aidixi and ^b^Akalux have not received FDA/EMA approval yet.

Abbreviations: ALL, acute lymphoblastic leukaemia; AML, acute myeloid leukaemia; APL, acute promyelocytic leukaemia; ADCs, Antibody drug conjugates; BCL, B‐cell lymphoma; BCMA, B‐cell maturation antigen; BsAbs, bispecific antibodies; BiTEs, bispecific T‐cell engager; cHL, classical Hodgkin Lymphoma; CRS, cytokine release syndrome; DLBCL, diffuse large B‐ cell lymphoma; EGFR, epidermal growth factor receptor; FRα, folate receptor alpha; GPRC5D, G protein‐coupled receptor class C group 5 member D; GEJ, gastroesophageal junction; HSCT, haematopoietic stem cell transplant; HCL, hairy cell leukaemia; HGBL, high‐grade BCL; HR, hormone receptor; HER2, human epidermal growth factor receptor 2; HLA, human leukocyte antigen; ICANS, immune effector cell associated neurotoxicity syndrome; ILD, interstitial lung disease; LBCL, large B‐cell lymphoma; LVEF, left ventricular ejection fraction; MET, mesenchymal–epithelial transition factor; MRD, minimal residue disease; MF, mycosis fungoides; NSCLC, non‐small cell lung cancer; N/A, not applicable; NOS, not otherwise specified; pcALCL, primary cutaneous anaplastic large cell lymphoma; PML, progressive multifocal leukoencephalopathy; REMS, risk evaluation and mitigation strategy; SJS, Stevens–Johnson syndrome; sALCL, systemic anaplastic large cell lyphoma; TCL, T‐cell lymphoma; TEN, toxic epidermal necrolysis; TNBC, triple‐negative breast cancer; UC, urothelial cancer; VOD, veno‐occlusive disease.

^a^ Aidixi (10.1007/s40265‐021‐01614‐x) by NMPA (National Medical Products Administration of China).

^b^ Akalux (10.1080/2162402X.2020.1841393) is the world's first phototherapy drug approved by PMDA (Pharmaceuticals and Medical Devices Agency of Japan).

### 4.1 CD30 targeting

Brentuximab‐vedotin (BV) is a CD30‐targeting ADC coupled via a protease‐cleavable, valine‐citrulline peptide linker to the tubulin polymerization inhibitor, monomethyl auristatin (MMA)‐E (MMAE), vedotin. CD30 is a TNF‐α receptor family member expressed on activated T‐, B‐ and NK‐cells. CD30 expression is restricted to a small subset of activated T‐ and B‐cells, elevated in certain lymphoid and non‐lymphoid neoplasms. Combined action of CD30 and OX40 is shown to be crucial for CD4 T‐cell responses and CD8 effector T‐cell recruitment.^122^ Interestingly, some studies observe BV activity independent of CD30 expression and the objective response rate (ORR) was found close to 40% in cases with undetectable CD30. Potential off‐target bystander effects in regions with low or undetectable target expression could therefore be a cause for concern. Common side effects observed following BV treatment include fatigue, nausea, fever, poor appetite and peripheral sensory neuropathy. Evaluation of combination therapies with ipilimumab, nivolumab and chemotherapy to further optimize the safety and tolerability is a subject of active exploration.^123^, ^124^ The payload, MMAE causes cell cycle arrest, inducing apoptotic cell death due to tubulin disruption in target T cells, following CD30 internalization. All‐grade neurotoxicity is associated with ADCs employing potent auristatins such as MMAE. Neutropenia and severe peripheral neuropathy in MMAE‐based ADC could be attributed to disruption of microtubule function of bone marrow (BM) mitosis/neuronal interphase tubule function. Ocular toxicity was reported for MMAF ADCs, while thrombocytopaenia and hepatic toxicity were associated with emtansine (DM1).^125^ Despite belonging to the auristatin payload family, charge retention in MMAF was reported to promote intracellular accumulation within the corneal epithelium, unlike the ability of MMAE to diffuse out following linker cleavage and release, due to the hydrophobic nature.^126^ Thus, ADC‐associated irAEs are additionally determined by properties of the toxic payload and linker characteristics used for conjugation. Second‐generation cleavable maleimide‐based linkers used in BV and polatuzumab‐vedotin (PV) show improvement over earlier ADC approaches, however, can still undergo premature release causing side effects such as neutropenia and neuropathy.^127^

### 4.2 CD22 targeting

CD22 is a Siglec family recycling receptor, expressed on both normal and malignant B‐cells. Abs targeted to CD22 are rapidly internalized and transported to endo‐lysosomal compartments where the cytotoxic moiety is released. Cleavable linkers or Abs dissociating in a pH‐dependent manner allow for the optimization of CD22‐based ADCs.^128^ Notable AEs upon CD22‐targeting, inotuzumab‐ozogamicin (INO) treatment include thrombocytopenia, anaemia, transaminitis, hepatotoxicity and life‐threatening veno‐occlusive disease (VOD). INO was found effective in a preclinical human pre‐B‐acute lymphoblastic leukaemia xenograft mouse model. The experimental mice remained healthy and active and slight aberrations observed in total bilirubin content were not attributable to treatment effects;^129^ however as indicated on the FDA label boxed warning, hepatoxicity was reported as a serious concern in clinical trials. CD22‐Fv fragment, fused to Pseudomonas endotoxin A (PE38), moxetumomab pasudotox, Lumoxiti, earlier approved in 2018 for specific cases of relapsed or refractory hairy cell leukaemia was found to cause AEs including hypertension; febrile neutropenia; haemolytic uraemic syndrome (HUS) and capillary leak syndrome (CLS), attributed to the superantigen PE38, which were indicated as FDA label boxed warnings. Lumoxiti withdrawal in 2023 was attributed to the low clinical uptake due to alternative treatment options and possibly due to the complex administration, toxicity monitoring and prophylaxis requirements following the administration (https://professionals.optumrx.com/content/dam/optum3/professional‐optumrx/news/rxnews/drug‐withdrawls/drugwithdrawal_lumoxiti_2023‐0117.pdf). Careful monitoring of blood parameters for cell number alterations and/or bleeding symptoms, alongside dosing adjustments, are crucial monitoring measures adopted in the clinical protocols.

### 4.3 CD33 targeting

Gemtuzumab‐ozogamicin (GO) was the first CD33 targeting ADC initially withdrawn due to substantial morbidity and mortality by VOD. GO was re‐approved in 2017, at a threefold lower dose that improved tolerability in CD33‐positive acute myeloid leukaemia (AML) or relapsed/refractory AML patients.^130^ GO is designed with the same cytotoxic moiety as INO and has been evaluated both as a single agent and combination with chemotherapy.^131^

GO‐based irAE could be attributed to widespread CD33 expression on normal myeloid progenitor cells and monocytes leading to severe neutropenia (90% of patients) which in turn predisposes the patient to severe bacterial and fungal infections, at times resulting in long periods of hospitalization and risk of life. CD33 expression on hepatocytes including hepatic sinusoidal endothelial cells may be causative for moderate‐to‐severe hepatic impairment and progression to fatal hepatic VOD. Grade 3 or 4 haematological and non‐haematological toxicities lead to infections and mucositis, in addition to fevers, chills, nausea and vomiting, dyspnoea, hypertension, hypotension and asthenia.^132^ The calicheamicin payload, ozogamicin, used in both CD33 and CD22 targeting ADCs is a DNA minor groove intercalator causing DNA breakage and apoptosis in target cells. Calicheamicin could be inferred as contributing to hepatotoxicity observed in GO and INO ADCs, since cytotoxicity could in part be imparted by the common payload. Bystander effects on neighbouring cells may occur due to payload diffusion to extracellular space. Ab‐calicheamicin conjugates were shown to cause alterations in hepatic architecture with platelet accumulation and loss of sinusoidal cells in some non‐human primate (NHP) models.^133^

Several CD33‐based therapeutic molecules currently being evaluated for AML are in various phases of clinical development.^134^, ^135^ Additional research is needed to understand the undesirable toxic capacity of CD33 targeting and correlation with genetic polymorphisms in CD33 as a means of patient stratification. The utility of CD33 expression on monocytic cells has gained interest as a predictive biomarker to determine responsiveness to ICI therapy in some cancers.^136^ The most concerning ‘on‐target/off‐tumour’ AEs require careful evaluation starting with the identification of patient subsets that may benefit from treatment to molecular design coupled with dose refinements for improving treatment outcomes.

### 4.4 CD79b targeting

PV is the first in class CD79b targeting ADC conjugated to vedotin, the same cytotoxic agent as the CD30‐targeting BV. CD79b is a transmembrane, signalling component of the B‐cell receptor expressed exclusively on normal and malignant B‐cells, but not on plasma and other haematologic cells making it an attractive target antigen.^137^ Safety and efficacy evaluation of PV in combination with bendamustine and rituximab observed low blood counts, peripheral neuropathy, fatigue, diarrhoea, fever, low appetite and pneumonia as the most common AEs.^121^ Close monitoring for early recognition of associated symptoms and signs are recommended for this treatment option.

### 4.5 HER2 targeting

Trastuzumab‐emtansine (T‐DM1) is an ADC indicated for the treatment of HER2‐positive metastatic breast cancer, consisting of humanized Ab, conjugated to a cytotoxic microtubule inhibiting maytansinoid, DM‐1, leading to cell cycle arrest and cell death. Stem cell‐like breast cancer cells are additionally shown to be targeted by T‐DM1, contributing to clinical efficacy.^138^ Nectin‐4, EGFR, Trop‐2, tissue factor, CD19, and folate receptor‐a have been added to the repository of druggable targets and recent approvals (Table 4), further supporting the development of therapies targeting these molecules.^139^, ^140^, ^141^ Ocular toxicity including changes in corneal epithelium and conjunctiva leading to vision changes, severe loss of vision and corneal ulceration were listed as significant adverse events reported in the FDA boxed warning for BCMA‐targeting belantamab mafodotin, Blenrep (approved in 2020 for specific cases of relapsed or refractory multiple myeloma) was withdrawn in late 2023.^142^ Proposed mechanisms contributing to the ocular toxicity include premature payload release due to extracellular cleavage of linker, subsequent intracellular metabolism of linker‐cytotoxic payload and cellular uptake mediated by Fc‐receptors.^143^ Thus, the toxic payload may additionally determine ADC‐associated irAEs and optimizing conjugate stability by focusing on linker chemistry could mitigate off‐tumour/ on‐target toxicities observed with these ADC classes. Preclinical evaluations of ADCs have not reported ocular toxicities and extending preclinical assessments to incorporate comprehensive assessments including ophthalmic examinations in multiple species and development of organ‐specific model systems will better address associated toxicity and improve translatability.^126^, ^144^

By design, ADCs allow for more targeted chemotherapy; however, improved toxicity profile, crucial to the favourable therapeutic index, requires new conjugation strategies and novel payloads in addition to therapeutic target selection. Predominantly used cytotoxic agents in ADC design directly disrupt important cellular machineries including DNA replication, transcription or tubulin polymerization. An important characteristic for payload design index is achieving high cytotoxic potency. Development of site‐specific conjugation methods, yielding ADCs with better defined and higher drug‐to‐Ab ratios, will prove beneficial in terms of larger therapeutic windows and are likely to be better tolerated than randomly conjugated ADCs. Introducing functional groups for Ab conjugation to improve solubility and stability in aqueous solution supports efficient conjugation and in vivo pharmacokinetic profiles. The past decade has seen many key improvements in the design of cytotoxic payloads and linker technologies essential to increase homogeneity and potency of ADCs. Continued advancements in biochemical design of cytotoxic payloads and conjugation strategies are crucial to driving creation of novel and safer ADCs.^145^, ^146^, ^147^

## 5 RISK ASSESSMENT FOR irAEs

Occurrence of immunological toxicities has the hampered clinical success of several Ab‐based cancer therapies and stunted multiple pipelines of drug development. Moreover, the burden of morbidity associated with immune‐mediated (IM) effects of clinically approved immunotherapies remains a significant clinical challenge. Kinetics of AE onset vary with individual patients and reactivity symptoms can appear soon after or several months following treatment initiation.^20^ Clinical irAE management presents challenges due to inherent complexity of diagnosis and heterogeneity of presentation in patients, often requiring multidisciplinary collaboration.^148^ Despite the availability of clinical interventions to resolve milder AEs, severe side effects can lead to treatment discontinuation or even fatal outcomes in patients. Chronic symptoms requiring immunosuppression or hormonal treatments maybe additionally experienced in some patients. There is a large body of specialized literature, focussing on the mechanism, presentation and clinical management of irAEs to optimize patient stratification and care;^79^, ^149^ however, these are beyond the scope of the current review. Importantly, further work is underway to improve guidance in the early recognition and management of irAE by specialist groups including the Society for Immunotherapy of Cancer (SITC) Toxicity Management Working Group.^150^

In terms of drug development, building robust, reliable tools and approaches predictive of response in patients will streamline the progress of novel biomolecules in the clinical pathway. Especially in the context of Ab‐based therapies where CRS is a significant irAE, inclusion of CR assays (CRAs) in preclinical assessment can support the design and selection of safer candidates with a better outcome in clinical trials. In vitro and ex vivo CRAs utilize human peripheral blood mononuclear cells (PBMCs) and whole blood (WB) as tools for safety assessment and help overcome challenges of complex animal models. We and others have shown that differences in solid versus aqueous phase presentation of the therapeutic and frequency/ proportion of immune responder cells present in WB or PBMC preparations are crucial response determinants.^151^, ^152^ Testing with a sufficiently inclusive experimental group size is imperative for better hazard identification and clinical success. Demonstration of intra‐laboratory and interlaboratory comparability in measurements is crucial for robust bridging of analytical readouts in preclinical assays. Harmonized identification of qualitative and quantitative determinants of responses leading to CRS development in patients will enable prediction and reliable risk identification during preclinical safety assessment. The development of assay standards and reference materials (RM) supports this process by ensuring uniformity in measurements and assay readouts to enable confidence in the validity of data generated during different evaluations conducted across sites and allows harmonization of data reporting by setting clear benchmarks to control assay performance and validation. Our laboratory recently developed a reference Ab panel including three positive control Abs (human anti‐CD52, mouse anti‐CD3 and human anti‐CD28 mAb super‐agonist (SA), known to induce different intensity of CRS in the clinic, and the respective isotype‐matched negative controls (human IgG1, mouse IgG2a and human IgG4). The relative capacity of these control Abs to stimulate the release of IFN‐γ, IL‐2, TNF‐α and IL‐6 in vitro was evaluated in an international collaborative study involving 11 laboratories.^153^ Due to the diversity of CRA platforms used by participants (solid vs aqueous phase, whole blood vs isolated cell types), a high interlaboratory variability was observed in terms of cytokine levels induced by the three positive control mAbs, but similar response patterns were observed across laboratories between the similar type of assays. The reference panel thus provides a benchmark for assay performance, validity, and variability to aid comparability and harmonization of CRAs performed in preclinical settings and will support improved CRS risk prediction in preclinical assessments.^153^ Expanding the scope of biological assay research towards the development of assays using differentiated cells representative of a wider immunological cell repertoire including elements of the TME such as macrophages, dendritic cells, neutrophils, NK and regulatory cells could identify effects on subset differentiation, polarization or suppression that may occur following in vivo administration of a biotherapeutic. Current preclinical in vitro models perform well for hazard identification, however, are not designed to predict the risk of cytokine responses at clinically relevant exposures. Therefore, harmonized translational models for characterizing dose‐dependent CR and immunologically modelling toxicity profiles will be valuable.

Animal models for preclinical safety assessment use immunodeficient and immunocompetent mouse models for proof‐of‐concept studies while complex animal models with rats, dogs, pigs, rabbits and NHP systems provide higher order models for preclinical safety and efficacy evaluations. Evaluation of absorption, distribution, metabolism and elimination (ADME) properties of oncological therapies, especially ADCs in Sprague Dawley rats can provide significant insight into catabolism and nonspecific distribution that could lead to payload‐associated toxicity and off‐target hepatic or renal impairment observed in patients.^154^ Use of NHP models such as cynomolgus monkey can provide extended information, however, could prove limited in utility due to the expense, amounts of Ab required for evaluation. More importantly these models rarely completely recapitulate the entire spectrum of overt toxicities related to lymphocyte activation, though CR potential or inflammatory responses can be measured in some settings. The CD28‐SA, TGN1412, showed a good safety profile in NHP models; however, the clinical trial had to be stopped due to life‐threatening cytokine storm occurring in all six healthy participants.^155^ Further studies, to understand the reasons behind the TGN1412 clinical trial failure, revealed that CD28 is highly expressed in human CD4^+^ effector memory T‐cells, but not in cynomolgus or rhesus macaques employed in preclinical testing.^156^, ^157^ In addition, preclinical in vitro assays employing human cells stimulated with TGN1412 in aqueous phase did not replicate the in vivo presentation that involves coreceptor cross‐linking at the immunological synapse.^158^ TGN1412 stimulation resulted in extensive IL‐2 and IFN‐γ release from human cells driving the cytokine storm in the clinical trial participants, an effect that could not be identified during earlier preclinical testing.^159^ Thus, the establishment of clinical safety in non‐human models may not always be representative of immunological response triggered in humans due to differences in repertoire of responder cells, response kinetics and target molecule. Nonetheless, studies evaluating the safety of ipilimumab and nivolumab combined therapy in cynomolgus macaques observed dose‐dependent immune‐related GI inflammation, an effect that was not observed when testing the Abs as single agents^160^ and recapitulation of clinically significant irAEs including monocytic infiltration in multiple organs and ICI‐associated myocarditis in these models.^161^ These studies highlight the importance and utility of relevant and multiple testing assays in both in vitro and in vivo systems to ascertain preclinical safety and capture the spectrum of overt toxicities that maybe encountered in the human system.^153^, ^159^ Comprehensive evaluations could markedly improve the safety of phase I clinical trials and enable drug development by identifying safer formulations at preclinical stage. The promising performance of several ADCs in preclinical research was not found to translate into clinical outcomes.^162^, ^163^ Therefore, future work in assay development and harmonizing translational models to adequately model immunological toxicity profiles will be valuable.

Immunocompetent gene knockout (GKO) mouse models are invaluable systems for the assessment of cancer immunotherapies. Novel models, such as CTLA‐4^+/−^ Pdcd1^−/−^ mice as a mechanistic model for ICI‐associated cardiovascular toxicities such as myocarditis, help identify strategies for interventional clinical AE management.^164^ Prominent differences from the human immune system (HIS) have led to focus on development of suitable humanized mice to model therapeutic benefit/ risk outcomes observed in clinical settings.^165^ HIS‐mouse models expand the scope of in vitro CRAs to help gain better insights on the biological complexity of human anti‐tumour response and support the safe and broad use of Ab‐based oncology therapeutics.^166^, ^167^, ^168^ Humanized mouse models rely on allogeneic lymphocytes that could be derived from BM, cord blood‐derived pluripotent human stem cells (HSCs), blood, foetal liver or PBMCs for immune reconstitution.^169^, ^170^ Reconstituted HIS‐mouse models recapitulate important features of the dynamics of human immune response generation and could therefore serve to potentially bridge in vitro evaluations and clinical outcomes. Several aspects including the ability to adequately represent relevant immune cell subsets such as innate lymphoid cells and neutrophils and model complexities of physiological responses in patients, however, need careful consideration while selecting the model for immuno‐oncological testing.^171^, ^172^ Recent developments coupling tumour engraftment in humanized mice have paved way for improved next‐generation in vivo model systems to support our understanding of immune‐oncological responses that maybe encountered in patients following administration of biological therapies.^173^, ^174^, ^175^ Autologous tumour engraftment models combine generation of cancer cell lines by transformation of primary fibroblast or induced pluripotent stem cell (iPSC)‐derived cells in conjunction with human foetal liver CD34^+^ and autologous foetal thymus tissue engraftment into immunocompromised SCID mice, generating a model referred to as BM, liver, thymus (Hu‐BLT) or humanized mice bearing autologous tumours (Hu‐AT) models.^176^, ^177^ Patient‐derived xenograft (PDX) models involving the use of patient‐derived tissues/explants simulate several patient‐specific oncological characteristics inclusive of heterogeneity and could likely develop as personalized therapy. However, practical considerations including patient consent and ethical approvals can complicate applicability in routine clinical practice.^178^ Optimized humanized mouse models offer a powerful tool to gain useful insight into effects of the treatment modalities targeting cancer and understand adverse outcomes, though due to the inherent complexity of human physiology, a single model may not address every aspect. Models and assays that adequately recapitulate the TME localized immune cell repertoire and replicate systemic off‐target effects that maybe encountered in the human system will support the clinical development of Ab‐based oncological therapeutics that are limited by toxicity. Evaluation of biological therapies in in vivo models can be inherently variable and differences in specificity and sensitivity of results could be encountered even while performing the same test by different operators or in a different facility. This makes standardization a challenging, but crucial input for harmonization and ensuring repeatability of data obtained in multi‐site comparisons. Results from our group evaluating the performance of a reference Ab panel^153^ (previously evaluated in vitro), in HSC or PBMC‐engrafted humanized mouse models, propose the inclusion of reference Abs in humanized mouse CRA model‐based risk evaluation for improved conformity during preclinical safety assessments (unpublished data). Mathematical and computational models enabling better understanding of pathological outcomes following treatment with Ab‐based cancer immunotherapeutics could aid improved therapeutic design for increased benefit to risk index and success of novel therapies in the clinic.^179^, ^180^, ^181^ Consolidating data obtained from in vitro, in silico and in vivo approaches in a uniform, reliable manner is essential to facilitate safer clinical translation of novel anti‐cancer therapies.

Organoid technology is emerging as a novel system to simulate in vivo tumour landscape and physiology. Patient‐derived organoid cultures can systematically recapitulate genetic diversity and histopathological features unique to the tumour thereby providing a means to investigate therapeutic interventions in a patient‐specific manner.^182^, ^183^ Advancements in three‐dimensional (3D) coculture systems such as organoids, spheroids and explants offer distinct advantages over two‐dimensional cocultures that are unable to adequately model tumour tissue architecture essential for reliable preclinical assessment. Organoids derived from specific areas of brain/neural tissue, retinal, kidney, lung, liver, GI and heart can be used to gain further insight into organ‐specific AE generation and support safer drug development.^184^ Biorepositories from cancer organoid cultures or tumouroids from patients with primary, metastatic or recurrent disease correlate with patient response and show promise as novel preclinical models to understand and predict individualistic response to treatment.^185^

Organ‐on‐a‐chip technology is another promising preclinical tool for recreating the complex microenvironment of human organs and interactions between different cell subtypes with the potential to simulate interaction between different organs.^186^ These new platforms could enable the safety assessment in tissues/organs susceptible to AEs.^187^, ^188^ Tunability of organ‐chip devices has proven useful for building complex 3D microenvironments representing vascular dissemination and tissue extravasation. By simulating cancer–immune interactions, immune organs‐on‐a‐chip similarly serve as important tools for preclinical drug testing, paving way for the development of effective therapeutics. In vitro hepatotoxicity assessment models constructing livers‐on‐a‐chip can in fact be combined with other organs‐on‐a‐chip such as heart, kidney and intestine to reconstruct dynamic interactions and aid the development of safer therapies. A recent study reporting a systematic assessment of performance of 870 liver‐chips shows that incorporating organ‐on‐a‐chip models will streamline the efficacy of safety evaluations in drug discovery pathways and improve confidence in progressing safer biologics to the clinic.^189^ Despite immense progress, the delivery of these approaches is extremely complex. Several steps starting with the availability and cellular quality of patient‐derived source material, suitability and optimization of assay conditions, and thresholds for validation in functional assays will benefit immensely from standards and RM for harmonization and establishment of unified, validated thresholds and critical quality attributes to guide clinical decisions and predict clinical success.^190^, ^191^ Focussed efforts to characterize performance and assess analytical validity of new tissue platforms against appropriate standards and RM will improve confidence in these technologies and advance regulatory acceptance.^192^

Guidelines from working groups such as ‘The response evaluation criteria in solid tumours’ (RECIST) ensure consistency in design and data collection in clinical trials for validation of immunological therapies, iRECIST. These efforts support the harmonization of data collection for measuring response to treatment using biomarkers (including imaging) and enable comparative assessment of results from different trials.^193^ Biomarker guidelines for quantifying toxicity and addressing adverse effects are in development.^194^ Published documents from the International Council for Harmonisation (ICH) of Technical Requirements for Pharmaceuticals for Human Use and FDA, guide analysis and preclinical risk evaluation. To exemplify, best practice guidelines for the assessment and management of IM liver injury secondary to ICI therapy were developed to aid drug development, describing pathological response, advising on patient stratification, conducting liver investigations prior to recruiting clinical trial patients and close monitoring with biochemical biomarkers (including liver function and other tests) to shield patients from severe and undesired hepatological effects.^195^ Comprehensive assessments to identify determinants maximizing desired on‐target effects and minimize therapy‐related side effects are derived based on the integration of several lines of clinical and nonclinical data. An iterative approach at the preclinical phase, incorporating data from existing clinical outcomes in patients, could thus propel informed, data‐driven, evidence‐based advancements.^196^

The International Consortium for Innovation and Quality in Pharmaceutical Development addresses and supports issues and challenges regarding implementation of micro‐physiological systems (MPS) in drug development as well.^197^ Clinical Data Interchange Standards Consortium (CDISC) and Clinical Data Acquisition Standards Harmonization (CDASH) address and identify important data characteristics including demographics and AEs, commonly observed in therapeutic research and clinical trials. Thus, standardization in efforts towards improving reporting of harms and stringent assessment of benefit/harm balance in clinical trials is significant for successful drug development. Systematic platforms such as the FDA‐safety information and AE‐reporting programme, MedWatch; the World Health Organization (WHO) global database, VigiBase (http://www.vigiaccess.org); EudraVigilance (http://www.ema.europa.eu/ema/index.jsp?curl=pages/regulation/general/general_content_000679.jsp&mid=WC0b01ac05800250b5); and UK‐MHRA yellow card scheme (https://yellowcard.mhra.gov.uk/) to identify and report post‐approval AEs of biological medicines can guide improved design and enhance patient safety. However, as these data are derived from voluntarily reported incidents, the population size is limited and uncertain, thereby proving to be a challenge in establishing a direct cause–effect relationship to a specific treatment. The accelerated rate of technological advancements and biological discoveries while offering several opportunities for streamlining preclinical and clinical research, make it additionally challenging to regulate future consequences of recent advancements brought in with artificial intelligence (AI) integration. Re‐focussing regulatory guidelines in a patient‐centric manner will be the key for confidence and interpretability of predictions and data derived from these developments.

## 6 CONCLUDING REMARKS

Treatment‐related toxicities form a major limitation in the clinical pathway of oncological interventions for existing and novel Ab‐based anti‐cancer therapies. Improving preclinical testing pathways is paramount to identify safer immunotherapies, and even enable modification for more favourable safety profile in order to avoid organ damage and future risks in clinical trials. In vitro and ex vivo assays using human cells may support hazard identification; however, the development of additional in vitro and in vivo methods to replicate patient physiology would be of great benefit. While functional assays may help identify and sometimes predict severity, patient responses observed following treatment are unique, heterogeneous and complex making it additionally challenging to recapitulate physiological outcomes at the level of an individual patient. Even with clinically approved immunotherapies, treatment may need to be stopped early in certain patients experiencing irAEs and treatment regimens and clinical management therefore could be specific for each patient. Pre‐treatment investigations and post‐treatment monitoring will remain a mainstay of the treatment pathway, with further work underway to improve on biomarkers and processes used for predicting and monitoring outcomes. Identification of biomarkers that will allow for better stratification of patients at high risk of severe, organ‐damaging or life‐threatening events prior to delivery of treatment will improve clinical success.

The emergence of novel technologies and automated assay platforms have streamlined functional assay development reducing turnaround times in sample processing and assay delivery. The development of standards and RM is crucial for assay harmonization and establishment of uniform, validated cut‐offs for hazard identification, thereby enabling reliable stratification of patient responses to specific treatment modalities in preclinical evaluation and clinical trials. These approaches will assist comprehensive assessments of determinants to maximize anti‐tumour efficacy and minimize therapy‐associated AEs. Consolidating patient data at preclinical phase could further propel informed advancements based on data‐driven evidence. Efforts on international harmonization will thus support and enable the broader scope of knowledge in our understanding of physiological responses, paving way for robust and reliable next‐generation therapeutics.

## AUTHOR CONTRIBUTIONS

**Deepa Rajagopal:** Writing – original draft (equal); writing – review and editing (equal). **Elliot MacLeod:** Writing – original draft (supporting); writing – review and editing (supporting). **Diana Corogeanu:** Writing – original draft (supporting); writing – review and editing (supporting). **Sandrine Vessillier:** Writing – original draft (equal); writing – review and editing (equal).

## CONFLICT OF INTEREST STATEMENT

The authors declare that they have no conflict of interests.

## Data Availability

Data sharing is not applicable to this article as no new data were created or analyzed in this study.
